# 

**Published:** 1934

**Authors:** 


### CONTENTS.

| | Page |
|---|---|
| The Association between Goitre and Atrophic Rhinitis. By E. Watson-Williams, M.C., Ch.M., F.R.C.S. | 89 |
| Focal Infection. By A. J. M. Wright, F.R.C.S. | 109 |
| The Diagnosis of Swellings of the Long Bones, with a Case of Periosteal Sarcoma of the Femur. By A. Wilfrid Admas, M.S., F.R.C.S. | 117 |
| The Expulsion of Bile. By R. J. Brocklehurst, D.M. | 131 |
| Reviews of Books | 139 |
| Editorial Note | 147 |
| Obituaries. Dr. John Lindesay Pearce, M.C. | 147 |
| Thomas Jones | 148 |
| Meetings of Societies | 149 |
| The Medical Library of the University of Bristol | 150 |
| Publications Received | 152 |
| Local Medical Notes | 153 |

